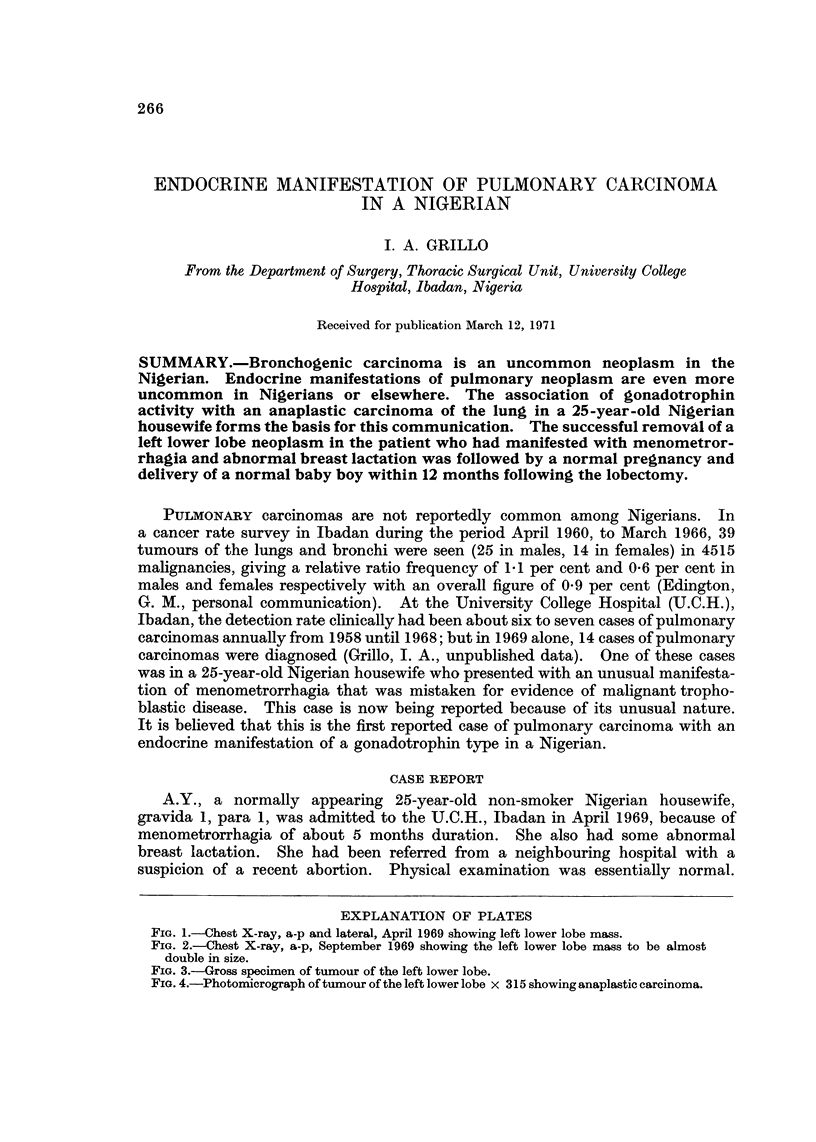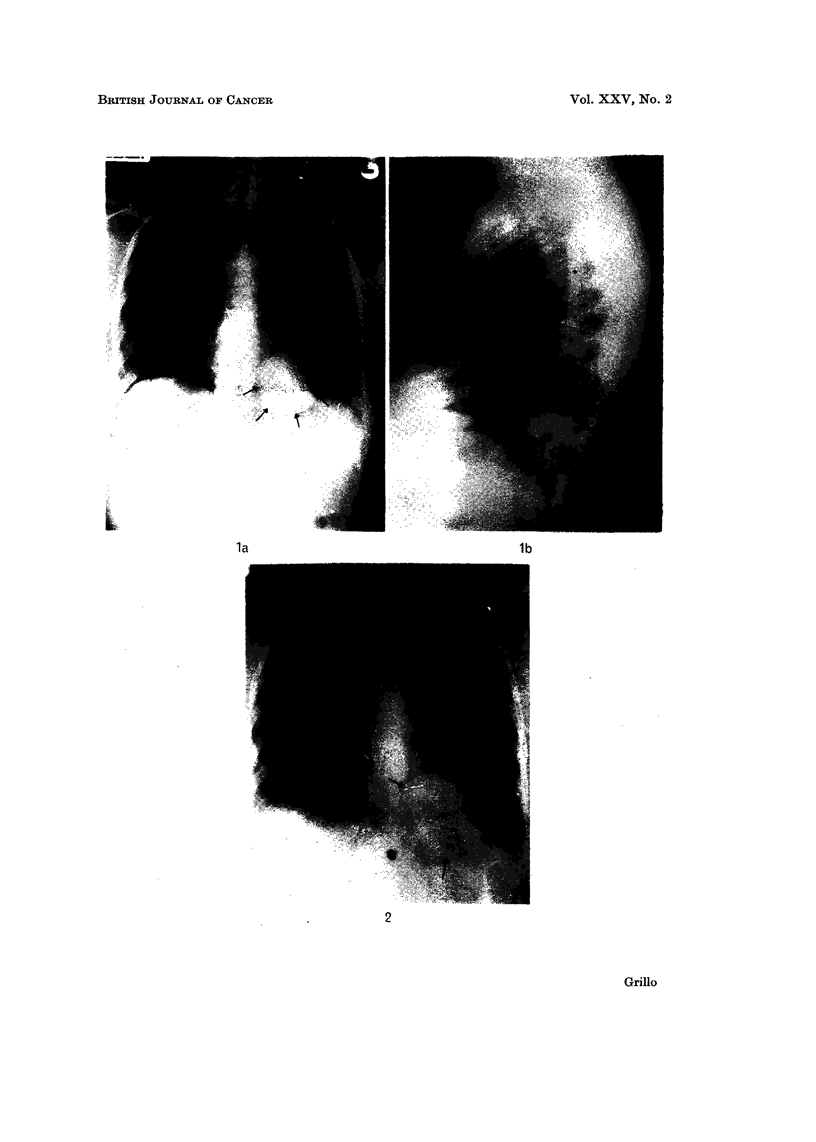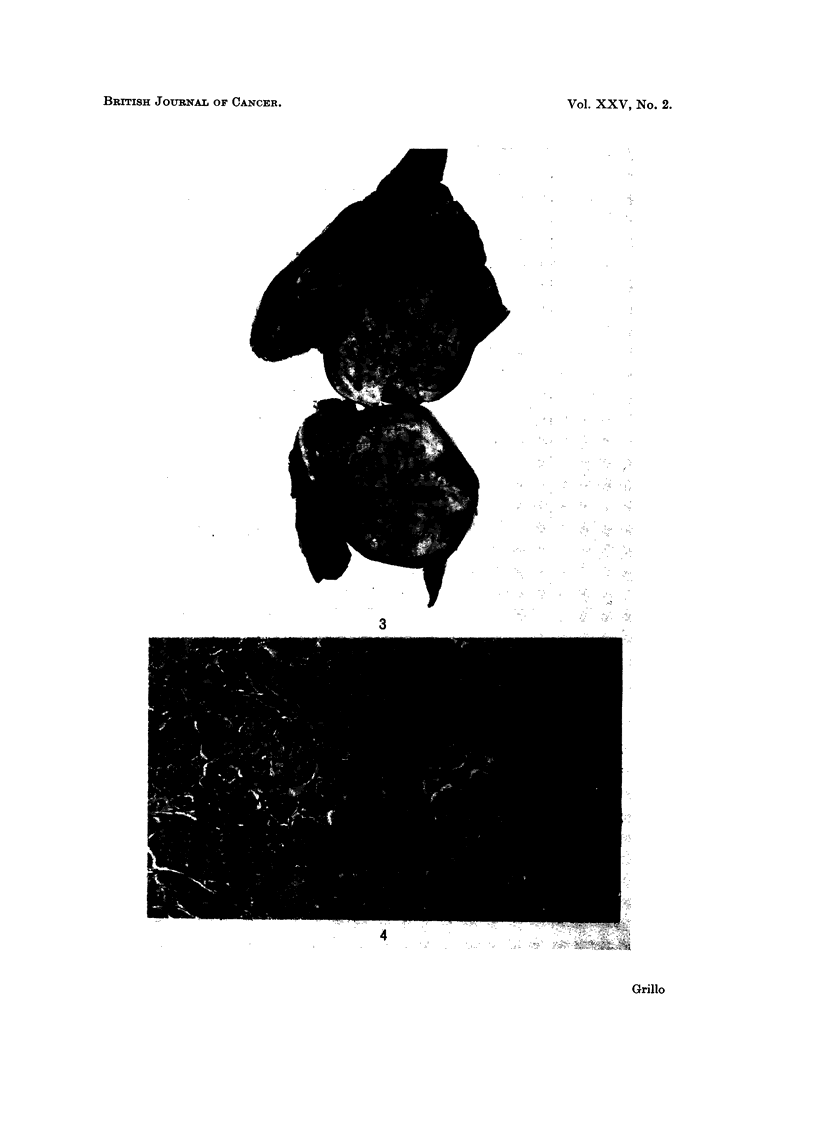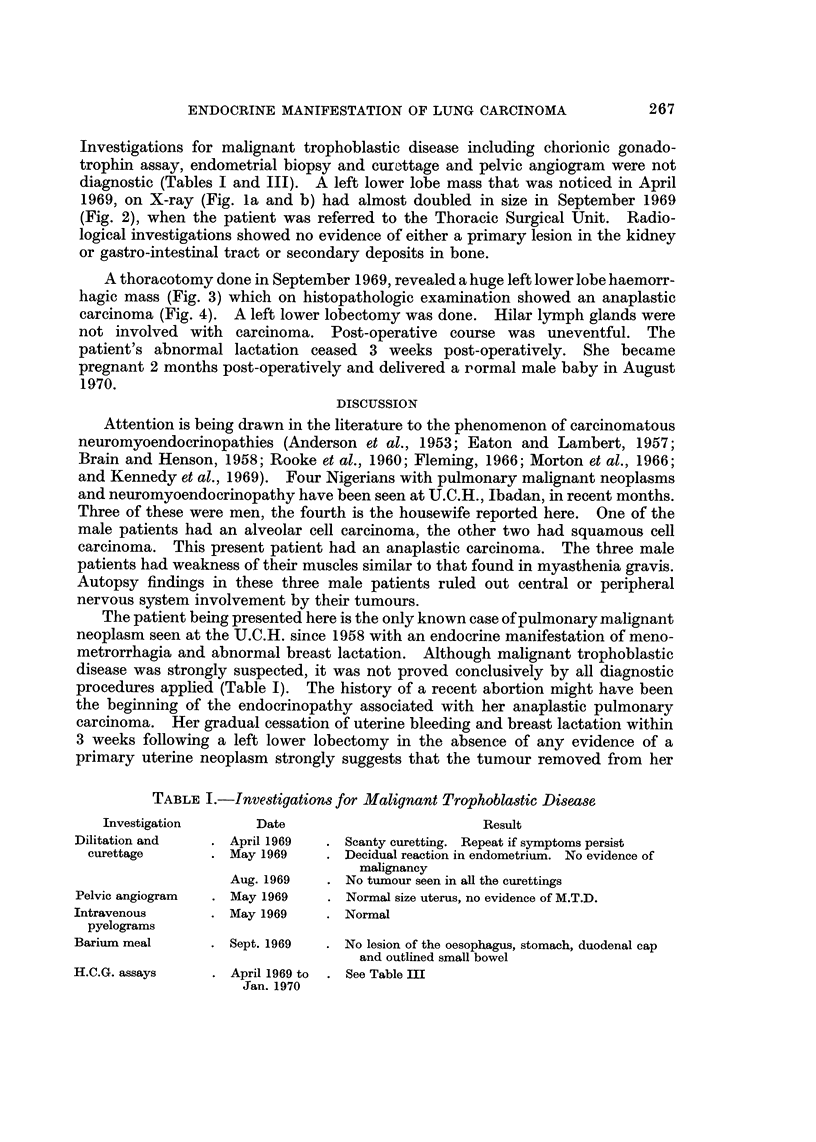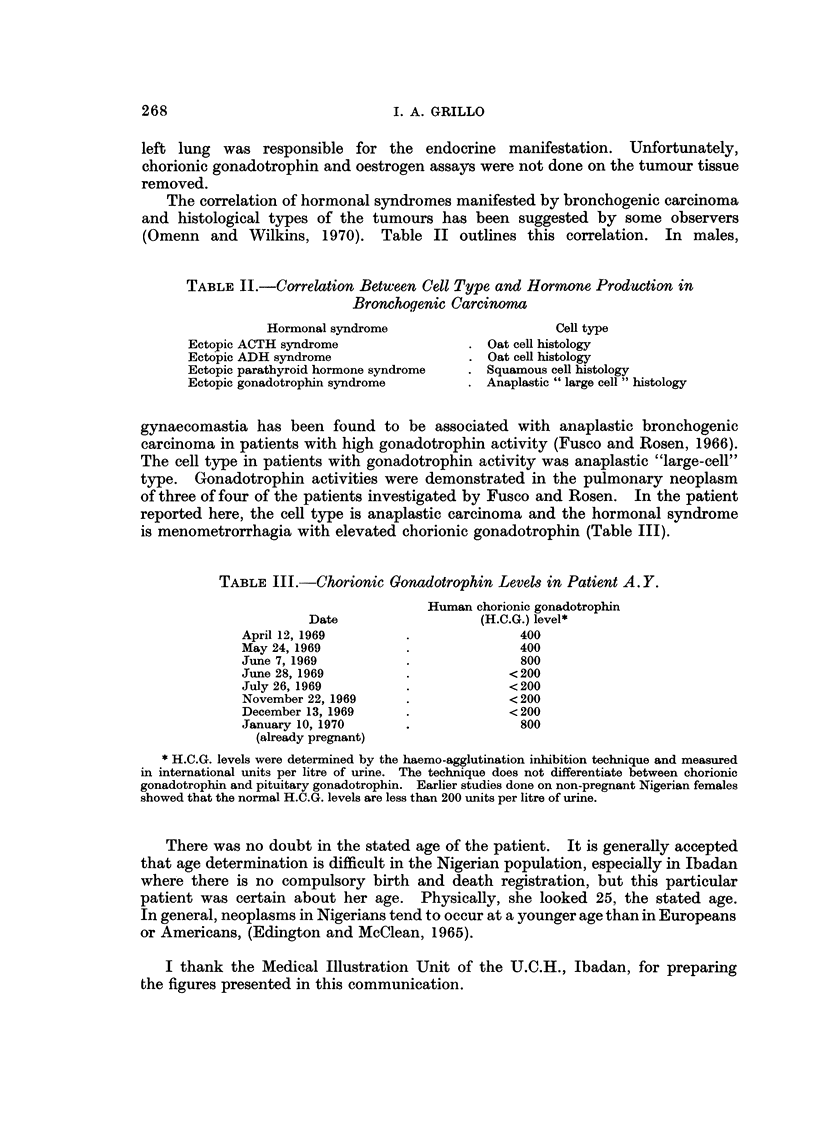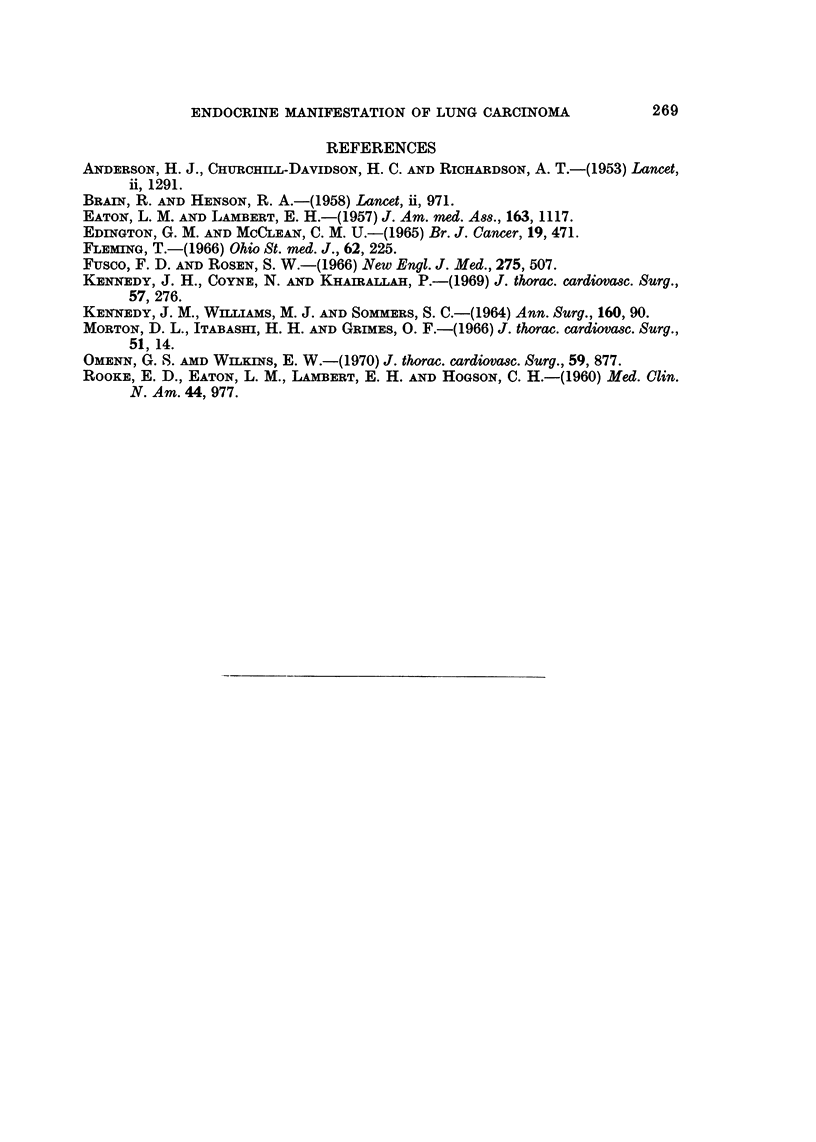# Endocrine Manifestation of Pulmonary Carcinoma in a Nigerian

**DOI:** 10.1038/bjc.1971.32

**Published:** 1971-06

**Authors:** I. A. Grillo

## Abstract

**Images:**


### ENDOCRINE MANIFESTATION OF PULMONARY CARCINOMA IN A NIGERIAN

I. A. GRILLO

*From the Department of Surgery, Thoracic Surgical Unit, University College Hospital, Ibadan, Nigeria*

Received for publication March 12, 1971

**SUMMARY.—Bronchogenic carcinoma is an uncommon neoplasm in the Nigerian. Endocrine manifestations of pulmonary neoplasm are even more uncommon in Nigerians or elsewhere. The association of gonadotrophin activity with an anaplastic carcinoma of the lung in a 25-year-old Nigerian housewife forms the basis for this communication. The successful removal of a left lower lobe neoplasm in the patient who had manifested with menometrorrhagia and abnormal breast lactation was followed by a normal pregnancy and delivery of a normal baby boy within 12 months following the lobectomy.**

PULMONARY carcinomas are not reportedly common among Nigerians. In a cancer rate survey in Ibadan during the period April 1960, to March 1966, 39 tumours of the lungs and bronchi were seen (25 in males, 14 in females) in 4515 malignancies, giving a relative ratio frequency of 1·1 per cent and 0·6 per cent in males and females respectively with an overall figure of 0·9 per cent (Edington, G. M., personal communication). At the University College Hospital (U.C.H.), Ibadan, the detection rate clinically had been about six to seven cases of pulmonary carcinomas annually from 1958 until 1968; but in 1969 alone, 14 cases of pulmonary carcinomas were diagnosed (Grillo, I. A., unpublished data). One of these cases was in a 25-year-old Nigerian housewife who presented with an unusual manifestation of menometrorrhagia that was mistaken for evidence of malignant trophoblastic disease. This case is now being reported because of its unusual nature. It is believed that this is the first reported case of pulmonary carcinoma with an endocrine manifestation of a gonadotrophin type in a Nigerian.

### CASE REPORT

A.Y., a normally appearing 25-year-old non-smoker Nigerian housewife, gravida 1, para 1, was admitted to the U.C.H., Ibadan in April 1969, because of menometrorrhagia of about 5 months duration. She also had some abnormal breast lactation. She had been referred from a neighbouring hospital with a suspicion of a recent abortion. Physical examination was essentially normal.

---

EXPLANATION OF PLATES

FIG. 1.—Chest X-ray, a-p and lateral, April 1969 showing left lower lobe mass.
FIG. 2.—Chest X-ray, a-p, September 1969 showing the left lower lobe mass to be almost double in size.
FIG. 3.—Gross specimen of tumour of the left lower lobe.
FIG. 4.—Photomicrograph of tumour of the left lower lobe × 315 showing anaplastic carcinoma.

1a

1b

2

Grillo

3

4

Grillo

Investigations for malignant trophoblastic disease including chorionic gonadotrophin assay, endometrial biopsy and curettage and pelvic angiogram were not diagnostic (Tables I and III). A left lower lobe mass that was noticed in April 1969, on X-ray (Fig. la and b) had almost doubled in size in September 1969 (Fig. 2), when the patient was referred to the Thoracic Surgical Unit. Radiological investigations showed no evidence of either a primary lesion in the kidney or gastro-intestinal tract or secondary deposits in bone.

A thoracotomy done in September 1969, revealed a huge left lower lobe haemorrhagic mass (Fig. 3) which on histopathologic examination showed an anaplastic carcinoma (Fig. 4). A left lower lobectomy was done. Hilar lymph glands were not involved with carcinoma. Post-operative course was uneventful. The patient's abnormal lactation ceased 3 weeks post-operatively. She became pregnant 2 months post-operatively and delivered a normal male baby in August 1970.

### DISCUSSION

Attention is being drawn in the literature to the phenomenon of carcinomatous neuromyoendocrinopathies (Anderson *et al.*, 1953; Eaton and Lambert, 1957; Brain and Henson, 1958; Rooke *et al.*, 1960; Fleming, 1966; Morton *et al.*, 1966; and Kennedy *et al.*, 1969). Four Nigerians with pulmonary malignant neoplasms and neuromyoendocrinopathy have been seen at U.C.H., Ibadan, in recent months. Three of these were men, the fourth is the housewife reported here. One of the male patients had an alveolar cell carcinoma, the other two had squamous cell carcinoma. This present patient had an anaplastic carcinoma. The three male patients had weakness of their muscles similar to that found in myasthenia gravis. Autopsy findings in these three male patients ruled out central or peripheral nervous system involvement by their tumours.

The patient being presented here is the only known case of pulmonary malignant neoplasm seen at the U.C.H. since 1958 with an endocrine manifestation of menometrorrhagia and abnormal breast lactation. Although malignant trophoblastic disease was strongly suspected, it was not proved conclusively by all diagnostic procedures applied (Table I). The history of a recent abortion might have been the beginning of the endocrinopathy associated with her anaplastic pulmonary carcinoma. Her gradual cessation of uterine bleeding and breast lactation within 3 weeks following a left lower lobectomy in the absence of any evidence of a primary uterine neoplasm strongly suggests that the tumour removed from her

TABLE I.—*Investigations for Malignant Trophoblastic Disease*

| Investigation | Date | Result |
|---|---|---|
| Dilitation and curettage | April 1969 | Scanty curetting. Repeat if symptoms persist |
| | May 1969 | Decidual reaction in endometrium. No evidence of malignancy |
| | Aug. 1969 | No tumour seen in all the curettings |
| Pelvic angiogram | May 1969 | Normal size uterus, no evidence of M.T.D. |
| Intravenous pyelograms | May 1969 | Normal |
| Barium meal | Sept. 1969 | No lesion of the oesophagus, stomach, duodenal cap and outlined small bowel |
| H.C.G. assays | April 1969 to Jan. 1970 | See Table III |

left lung was responsible for the endocrine manifestation. Unfortunately, chorionic gonadotrophin and oestrogen assays were not done on the tumour tissue removed.

The correlation of hormonal syndromes manifested by bronchogenic carcinoma and histological types of the tumours has been suggested by some observers (Omenn and Wilkins, 1970). Table II outlines this correlation. In males,

TABLE II.—*Correlation Between Cell Type and Hormone Production in Bronchogenic Carcinoma*

| Hormonal syndrome | Cell type |
|---|---|
| Ectopic ACTH syndrome | Oat cell histology |
| Ectopic ADH syndrome | Oat cell histology |
| Ectopic parathyroid hormone syndrome | Squamous cell histology |
| Ectopic gonadotrophin syndrome | Anaplastic " large cell " histology |

gynaecomastia has been found to be associated with anaplastic bronchogenic carcinoma in patients with high gonadotrophin activity (Fusco and Rosen, 1966). The cell type in patients with gonadotrophin activity was anaplastic "large-cell" type. Gonadotrophin activities were demonstrated in the pulmonary neoplasm of three of four of the patients investigated by Fusco and Rosen. In the patient reported here, the cell type is anaplastic carcinoma and the hormonal syndrome is menometrorrhagia with elevated chorionic gonadotrophin (Table III).

TABLE III.—*Chorionic Gonadotrophin Levels in Patient A.Y.*

| Date | Human chorionic gonadotrophin (H.C.G.) level* |
|---|---|
| April 12, 1969 | 400 |
| May 24, 1969 | 400 |
| June 7, 1969 | 800 |
| June 28, 1969 | $<200$ |
| July 26, 1969 | $<200$ |
| November 22, 1969 | $<200$ |
| December 13, 1969 | $<200$ |
| January 10, 1970 (already pregnant) | 800 |

* H.C.G. levels were determined by the haemo-agglutination inhibition technique and measured in international units per litre of urine. The technique does not differentiate between chorionic gonadotrophin and pituitary gonadotrophin. Earlier studies done on non-pregnant Nigerian females showed that the normal H.C.G. levels are less than 200 units per litre of urine.

There was no doubt in the stated age of the patient. It is generally accepted that age determination is difficult in the Nigerian population, especially in Ibadan where there is no compulsory birth and death registration, but this particular patient was certain about her age. Physically, she looked 25, the stated age. In general, neoplasms in Nigerians tend to occur at a younger age than in Europeans or Americans, (Edington and McClean, 1965).

I thank the Medical Illustration Unit of the U.C.H., Ibadan, for preparing the figures presented in this communication.

### REFERENCES

ANDERSON, H. J., CHURCHILL-DAVIDSON, H. C. AND RICHARDSON, A. T.—(1953) *Lancet*, ii, 1291.
BRAIN, R. AND HENSON, R. A.—(1958) *Lancet*, ii, 971.
EATON, L. M. AND LAMBERT, E. H.—(1957) *J. Am. med. Ass.*, **163**, 1117.
EDINGTON, G. M. AND McCLEAN, C. M. U.—(1965) *Br. J. Cancer*, **19**, 471.
FLEMING, T.—(1966) *Ohio St. med. J.*, **62**, 225.
FUSCO, F. D. AND ROSEN, S. W.—(1966) *New Engl. J. Med.*, **275**, 507.
KENNEDY, J. H., COYNE, N. AND KHAIRALLAH, P.—(1969) *J. thorac. cardiovasc. Surg.*, **57**, 276.
KENNEDY, J. M., WILLIAMS, M. J. AND SOMMERS, S. C.—(1964) *Ann. Surg.*, **160**, 90.
MORTON, D. L., ITABASHI, H. H. AND GRIMES, O. F.—(1966) *J. thorac. cardiovasc. Surg.*, **51**, 14.
OMENN, G. S. AMD WILKINS, E. W.—(1970) *J. thorac. cardiovasc. Surg.*, **59**, 877.
ROOKE, E. D., EATON, L. M., LAMBERT, E. H. AND HOGSON, C. H.—(1960) *Med. Clin. N. Am.* **44**, 977.